# Insights into the stereoisomerism of dihydroquercetin: analytical and pharmacological aspects

**DOI:** 10.3389/fchem.2024.1439167

**Published:** 2024-07-05

**Authors:** Roman P. Terekhov, Anastasiya D. Savina, Denis I. Pankov, Maria D. Korochkina, Amir Taldaev, Liubov M. Yakubovich, Sergey P. Zavadskiy, Anastasiya K. Zhevlakova, Irina A. Selivanova

**Affiliations:** ^1^ Nelyubin Institute of Pharmacy, Sechenov First Moscow State Medical University, Moscow, Russia; ^2^ Institute of Biomedical Chemistry, Moscow, Russia; ^3^ Research Center for Molecular Mechanisms of Aging and Aging-Related Diseases, Moscow Center for Advanced Studies, Moscow, Russia

**Keywords:** dihydroquercetin, flavonoid, stereoisomerism, optical activity, NMR spectroscopy, HPLC, pharmacodynamics, pharmacokinetics

## Abstract

Dihydroquercetin (DHQ) is a representative of flavonoids that is available on the market as a food supplement and registered as an active pharmaceutical ingredient. The structure of this compound is characterized by the presence of two chiral centers in positions 2 and 3 of the pyranone ring. Current regulatory documentation on DHQ lacks quantitative analysis of the stereoisomers of this flavanonol. This poses potential risks for consumers of DHQ-based dietary supplements and developers of new drugs. This review was conducted to systematize data on the pharmacology of DHQ stereoisomers and the possible methods of controlling them in promising chiral drugs. We found that relying on literature data of polarimetry for the identification of DHQ stereoisomers is currently impossible due to these heterogeneities. NMR spectroscopy allows to distinguishing between *trans*- and *cis*-DHQ using chemical shifts values. Only HPLC is currently characterized by sufficient enantioselectivity. Regarding pharmacology, the most active stereoisomer of DHQ should be identified, if the substituents in chiral centers both take part in binding with the biological target. The significant impact of stereochemical structure on the pharmacokinetics of DHQ isomers was reported. The question about these toxicity of these compounds remains open. The results of the conducted review of scientific literature indicate the necessity of revising the pharmacology of DHQ taking into account its stereoisomerism.

## 1 Introduction

Throughout the history of pharmaceutical science, herbal materials have been the focus of investigators (Bokov and Samylina, 2015; Bokov et al., 2020; Fitzgerald et al., 2020; Giannenas et al., 2020). Nowadays, natural compounds continue to be promising objects for drug development (Du et al., 2020; Shikov et al., 2021; Savelyeva et al., 2022; Rehman et al., 2023; Yang et al., 2023). Newman and Cragg (2020) reported that from 1981 to 2019 there was 1881 new remedies, which were approved for medical application, and the 22.9% of them are of natural origin. Phytotherapy plays an important role in the treatment of cardiovascular (Kirichenko et al., 2020; Archakova et al., 2022; Karahan et al., 2022), immune (Babich et al., 2020; Ligacheva et al., 2022; Abdugafurova et al., 2024), cancer (Medvedev et al., 2020; Kitaeva et al., 2022; Jia-Xing et al., 2023), and infectious diseases (Gorlenko et al., 2020; Luzhanin et al., 2022; Zrig, 2022). According to the WHO, herbal medications are the main choice in many regions of the world (Aware et al., 2022).

The number of natural compounds cannot be counted. In 1978, the Chemical Abstract Services contained information about 4,039,907 structures (Maugh, 1978), and now it is more than 279 million. In pharmaceutical science, natural compounds are classified based on their chemical structure to systematize their biological effects, extraction approaches, and chemical control.

Flavonoids are a group of natural polyphenols that widely occur in different parts of plants. They are derivatives of 1,3-diphenyl propane. The aglycones of flavonoids are characterized by acidic chemical centers, high solubility in alcohols and hot water, and high free radical scavenging capacity (Ilyasov et al., 2018; Ilyasov et al., 2020; Elapov et al., 2021; Agatonovic-Kustrin et al., 2022; Poluyanov et al., 2023). Traditionally, researchers associate the pharmacological effects of flavonoids with their antioxidant activity (Shilova et al., 2017; Shevelev et al., 2020; Mordvinov et al., 2021). However, currently, there is an increase in data that argues a contrary view: Flavonoids selectively bind to biological targets (Shubina et al., 2023; Taldaev et al., 2022B). With this knowledge in mind, the importance of flavonoids’ stereochemistry and the impact of different stereoisomers on human health is recognized.

**FIGURE 1:**
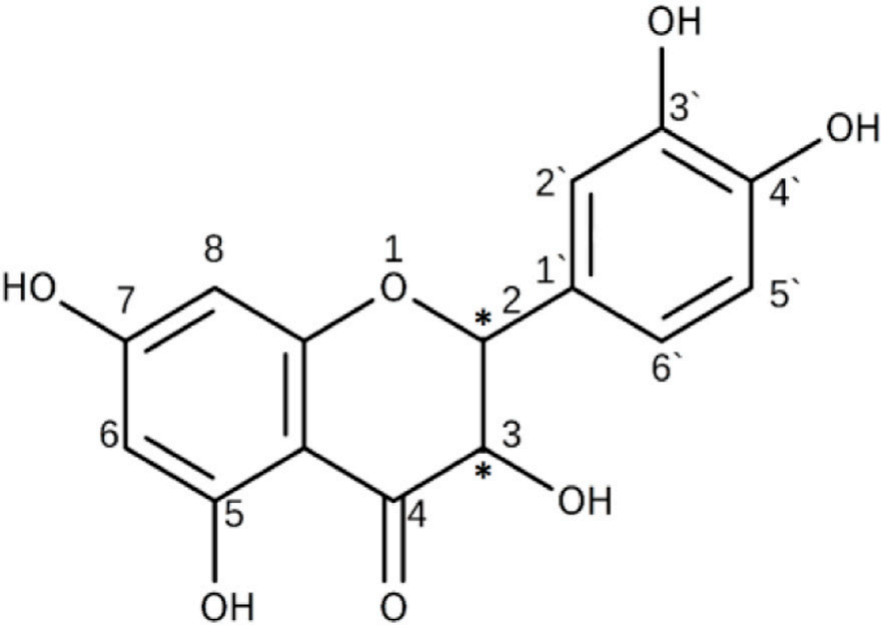
Structure of DHQ.

Dihydroquercetin (DHQ), also known as taxifolin, is a representative of flavonoids, specifically, flavanonols. The systematic IUPAC name of this compound is 2,3-dihydro-3,5,7-trihydroxy-2-(3,4-dihydroxyphenyl)-4H-1-benzopyran-4-one. The structure of this flavonoid is characterized by the presence of two chiral centers in positions 2 and 3 of the pyranone ring (Figure 1). This fact results in four possible stereoisomers of DHQ: a pair of enantiomers—(2*R*,3*R*)-DHQ and (2*S*,3*S*)-DHQ—are *trans*-DHQ, and another pair—(2*R*,3*S*)-DHQ and (2*S*,3*R*)-DHQ—are *cis*-DHQ. The pairs of *trans*- and *cis*-isomers are diastereomers of each other.

Diquertin^®^ is the first pharmaceutical product based on DHQ that has been registered in Russia. It was approved as an antioxidant in the treatment of acute pneumonia, chronic obstructive pulmonary disease, asthma, coronary heart disease, and supraventricular arrhythmia (Kolhir et al., 1998; Plotnikov et al., 2003). The reference sample of DHQ was developed to standardize the active pharmaceutical ingredient (API) of Diquertin^®^. Using chiroptical analytical methods, it was discovered that this sample consists of (2*R*,3*R*)-DHQ (Selivanova et al., 1999).

Currently, DHQ is registered as an API in the Russian Federation and the Republic of Kazakhstan. In the European Union, it is on the market as a food supplement. Various preclinical and clinical studies are ongoing in Russia, China, and Japan (Shinozaki et al., 2023; Taldaev et al., 2023; Choi et al., 2024).

Larix wood is a natural source for DHQ. Russia has a rich source of this raw material. Since 1970, 31 technologies related to the extraction of DHQ from natural sources have been patented in Russia (Table 1). The majority of these technologies involve the use of water and high temperatures, with 80.6% and 51.6% of the technologies describing them, respectively. In some cases, the resulting extract was purified by HPLC (SU 351847 A1, RU 2114631 C1, RU 2317093 C1, RU 2349 31 C1, RU 2359666 C2, and RU 2 435 766 C1). To increase the extraction yield, raw materials were mechanoactivated with a solid base (RU 2307834 C1 and RU 2 386 624 C2). In 2020, a patent for the extraction of DHQ with supercritical carbon dioxide was granted (RU 2 747 696 C1). Additionally, in 12.9% of cases, highly active acidic and basic agents are required. All these conditions can lead to isomerization of the flavonoid molecule (Kiehlmann and Li, 1995). Recent studies have confirmed these findings. For example, it was discovered that up to 3.7% of the molecules in commercially available DHQ samples have a *cis*-configuration (Terekhov et al., 2024A). Furthermore, spray drying was associated with a significant increase in the amount of the *cis*-isomer, reaching 9.5% (Taldaev et al., 2022A).

**TABLE 1:** Conditions of DHQ extraction technologies, patented in Russia.

Year	Solvents							Reagents		T, °C		Patent number
	H_2_O	Me_2_CO	MeOH	EtOH	*i*-PrOH	EtOAc	Others	Inorganic acid	Inorganic base	Min	Max	
1970	+	+	-	-	-	-	-	-	-	-	+100	SU 351847 A1
1992	+	-	-	-	-	+	-	-	-	-	+100	RU 2 000 797 C1
1993	+	-	-	-	-	+	hexane	-	-	-	+100	RU 2 034 559 C1
1994	+	+	-	-	-	-	-	-	-	-	+18	RU 2 038 094 C1
1995	-	-	-	-	-	+	hexane	-	-	-	+77	RU 2 082 425 C1
1996	+	+	-	+	-	-	-	-	-	-	+40	RU 2 091 076 C1
1997	+	+	-	-	-	-	-	-	-	-	+100	RU 2 114 631 C1
1998	+	-	-	-	-	+	hexane	-	-	+25	+98	RU 2 158 598 C2
	+	-	-	+	-	-	hexane	-	-	-	+40	RU 2 135 510 C1
	+	-	-	-	-	+	-	-	-	-	+70	RU 2 174 403 C2
2000	-	-	-	-	-	+	hexane	-	-	-	+40	RU 2 165 416 C1
2001	+	-	-	-	-	-	MeO*t*-Bu	-	-	+70	+92	RU 2 180 566 C1
	+	+	+	+	-	-	-	+	-	+40	+120	RU 2 184 561 C1
2002	+	-	-	+	-	-	-	-	-	+30	+40	RU 2 206 568 C1
	+	-	-	+	-	-	hexane	-	-	+30	+40	RU 2 211 836 C1
2003	+	-	-	-	-	-	MeO*t*-Bu	-	-	+70	+92	RU 2 233 858 C1
	+	+	+	+	-	+	-	-	-	-	+125	RU 2 255 750 C2
2004	-	-	-	-	-	-	hexane	-	-	-	+38	RU 2 252 220 C1
	-	+	-	-	-	+	urea	-	-	-	+120	RU 2 258 525 C1
2005	-	-	-	+	-	-	-	-	-	-	+25	RU 2 279 284 C1
2006	+	-	-	-	-	-	-	-	+	-	-	RU 2 307 834 C1
	+	-	+	+	-	-	-	-	-	0	+66	RU 2 317 093 C1
	+	+	+	+	-	+	-	+	-	-	+120	RU 2 318 528 C2
2007	+	-	-	-	+	+	-	-	-	-	+100	RU 2 346 941 C2
	+	-	-	+	-	-	MeO*t*-Bu	-	-	+20	+50	RU 2 330 677 C1
	+	-	+	+	+	-	-	-	-	+60	+60	RU 2 349 331 C1
	+	-	-	-	-	-	-	+	+	-	-	RU 2 386 624 C2
	+	+	-	+	+	-	-	-	-			RU 2 359 666 C2
2008	+	-	-	+	-	-	-	-	-	−40	+40	RU 2 372 095 C1
2010	+	-	-	+	-	-	-	-	-	+40	+50	RU 2 435 766 C1
2020	-	-	-	+	-	-	CO_2__liq_	-	-	+20	+31	RU 2 747 696 C1

However, current regulatory documentation on DHQ lacks indicators for the quantitative analysis of the stereoisomers of this flavanonol. Nevertheless, the Guidance document, published by FDA in 1992, prescribes to identify compounds with chiral centers and to know the quantitative isomeric composition of the material used in pharmacy (US Food and Drug Administration, 1992). The absence of proper control in this area poses potential risks for both consumers of DHQ-based dietary supplements and developers of new drugs. This literature review aimed to organize data on the pharmacology of DHQ stereoisomers and possible methods of controlling them in promising chiral drugs based on it.

## 2 General outlook on scientific landscape

**FIGURE 2:**
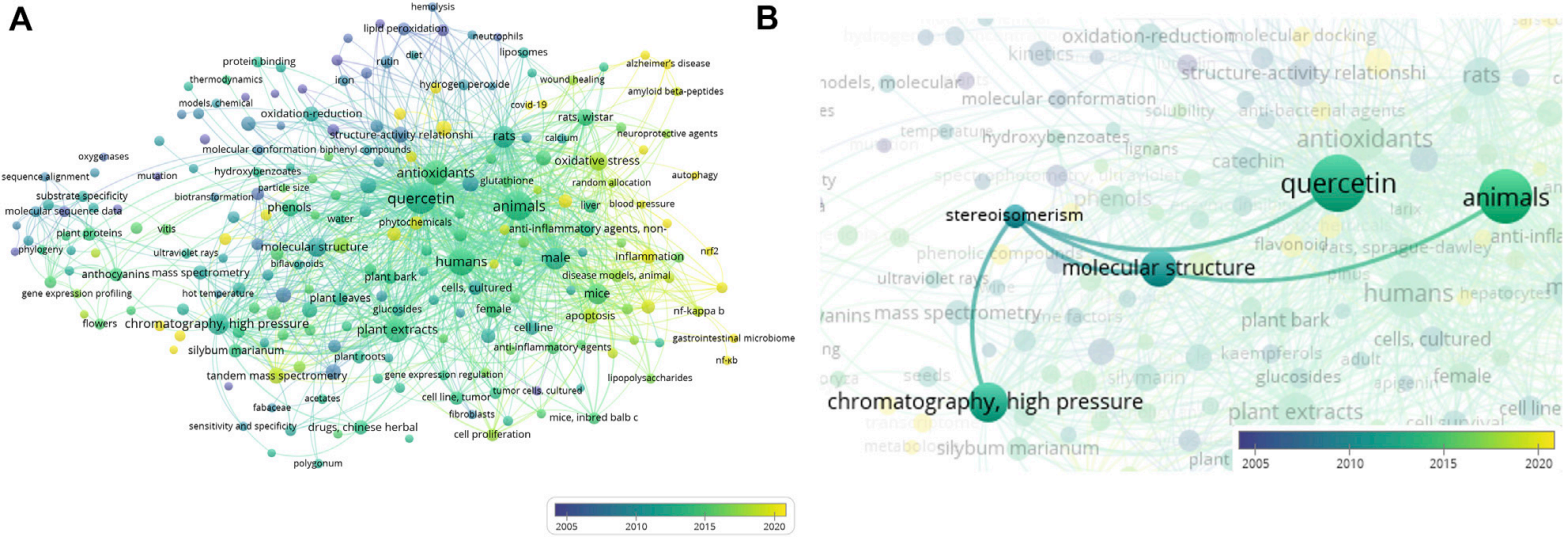
Bibliometric network of terms “taxifolin” and “dihydroquercetin”: **(A)** general view of network; **(B)** co-occurrence terms for “stereoisomerism”. Created by VOSviewer (Van Eck and Waltman, 2010; Van Eck and Waltman, 2011; Waltman and van Eck, 2013).

The literature data deposited in MEDLINE were used to build a bibliometric network based on keywords co-occurring with the terms “taxifolin” and “dihydroquercetin” in articles (Figure 2A).

The size of bubbles corresponds to the frequency of term mentions. The pseudocolor scale from blue to yellow reflects the novelty of articles from 2001 or earlier to the year 2024. What stands out in this figure is a pronounced trend in the area of DHQ investigation from the traditional analysis of plant extracts using standard chromatographic and spectral approaches to omics research (Mei et al., 2019; Mao et al., 2022). Additionally, these data confirm the increasing interest of investigators in the selective interaction of DHQ with different biological targets, such as nuclear factor erythroid 2-related factor 2 (NRF-2) (Turovskaya et al., 2019) and nuclear factor κ-light-chain-enhancer of activated B cells (NF-κB) (Akinmoladun et al., 2022). The opportunities for using DHQ in the therapy of socially significant diseases (Alzheimer’s disease and COVID-19) are increasingly in focus of research (Zhu et al., 2022; Hattori et al., 2023; Saxena et al., 2024). Furthermore, new computational methods of analysis have been implemented in research practice (Terekhov et al., 2019; Rudrapal et al., 2022; Ahmad et al., 2024).

Figure 2B shows a decrease in interest in the stereochemistry of DHQ over the last 10 years. In the latest articles, this topic is discussed in the context of the individual presence of DHQ enantiomers in plant extracts (Vega-Villa et al., 2009; Gaggeri et al., 2012). It is notable that in these studies, HPLC was used to separate the individual DHQ substances. Additionally, in some cases, chiroptical analytical methods, such as circular dichroism spectroscopy, were used to identify the configuration of the flavanonol molecule.

The next sections will focus on the major stereoselective analytical approaches used to control the configuration of DHQ molecules.

## 3 Analytical aspects

### 3.1 Polarimetry

Polarimetric analysis is the primary routine tool in the pharmacist’s arsenal for assessing the stereochemical composition of APIs.

Over the 76-year period of DHQ study, an impressive amount of data on the ability of solutions of different stereoisomers of this flavanonol to rotate the polarization plane of monochromatic light has been accumulated (Table 2).

**TABLE 2:** Bibliographic data on the polarimetric analysis of DHQ stereoisomers.

Stereoisomer	Solvent	Temperature °C	Concentration, mol/L	Angle of specific rotation, °	Reference
2*R*,3*R*	MeOH	16	0.10	+17.3	Sato et al. (2013)
		20	0.20	+12.7	Gaggeri et al. (2012)
		20	0.50	+19.4	Lundgren and Theander (1988)
		23	1.68	+22.0	Lee et al. (2003)
		24	0.10	+24.9	Yoon et al. (2020)
		25	0.50	+23.0	Kiehlmann and Li (1995)
		29	0.12	+22.2	Sato et al. (2013)
		30	0.50	+4.1	Imai et al. (2008)
		-	0.10	+19.0	Sato et al. (2013)
		-	0.35	+28.5	Kuroyanagi et al. (1982)
		-	0.45	+35.6	Inada et al. (1992)
	EtOH	18	0.56	+21.1	Kasai et al. (1988)
		25	0.75	+12.0	Sakurai and Okumura (1983)
	Me_2_CO	18	0.57	+24.6	Kasai et al. (1988)
		20	0.57	+17.3	Nonaka et al. (1987)
		-	0.57	+21.3	Ohmura et al. (2002)
	Me_2_CO + H_2_O	-	-	+26.0	Yoshida et al. (1989)
2*R*,3*S*	MeOH	20	0.50	−20.0	Lundgren and Theander (1988)
	Me_2_CO + H_2_O	20	0.13	−59.5	Nonaka et al. (1987)
2*S*,3*R*	MeOH	20	0.40	+30.5	Lundgren and Theander (1988)
	Me_2_CO	20	0.26	+58.8	Nonaka et al. (1987)
2*S*,3*S*	MeOH	16	0.10	−16.2	Sato et al. (2013)
		20	0.10	−15.0	Lundgren and Theander (1988)
	Me_2_CO	20	0.32	−20.6	Nonaka et al. (1987)

According to the literature data, alcohols and less frequently acetone are mainly used as solvents for the analyzed DHQ samples. The scientific community’s commitment to using these solvents is explained by DHQ’s solubility. Higher concentration is associated with better values of trueness, precision, and robustness. Limited solubility in combination with a small value of the specific rotation angle leads to a significant error in the results, especially in the absence of automated measurement. It is important to note that data obtained for samples in different solvents are not comparable due to the medium’s effect on the value of the rotation angle of the polarization plane of light.

**FIGURE 3:**
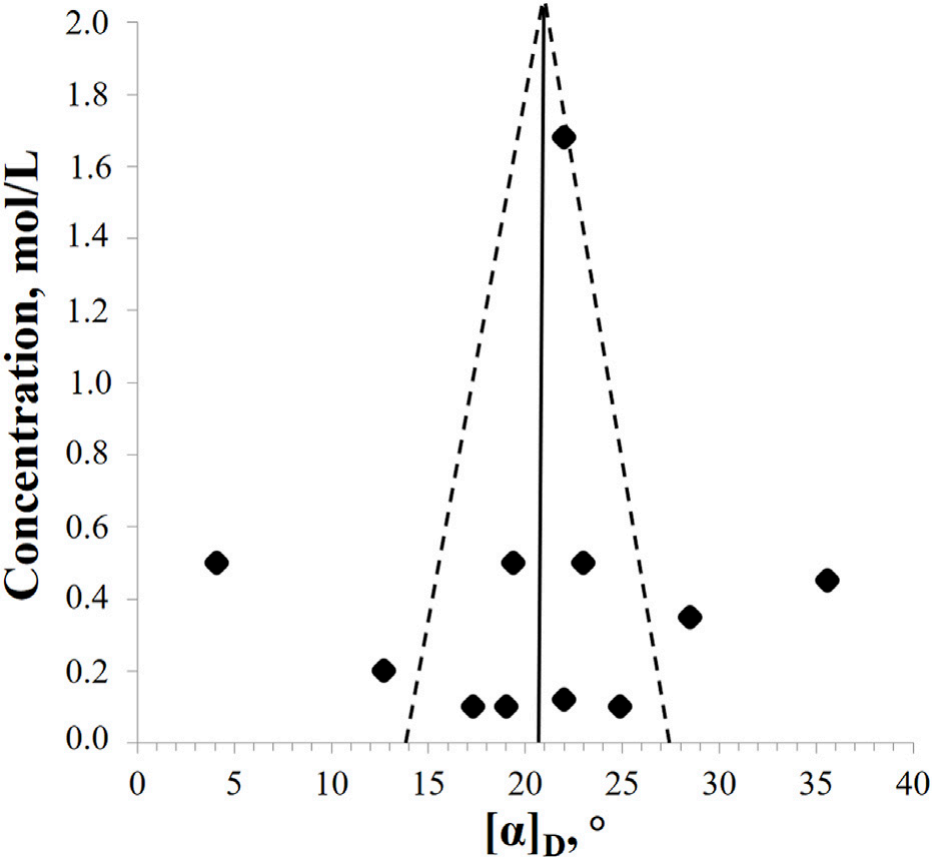
The funnel plot of relationship between (2*R*,3*R*)-DHQ concentrations in methanol solutions and values of specific ration angle (*p* = 0.01).

Methanol is most often used as a solvent. However, the available data are characterized by pronounced heterogeneity. Thus, for (2*R*,3*R*)-DHQ, the specific rotation values vary from + 4.1° to + 35.6°, with a mean value of + 20.8° ± 6.9°. No correlation was found between temperature and the measured value (*r* ^*2*^ = 0.0299). Based on the symmetry of the funnel plot, we can say that there are no publication errors or data defaults on the part of researchers (Figure 3). Obviously, the accuracy of the measurement increases with increasing concentration. However, the proportion of drop-out points remains high, so it is necessary to refine the available data by repeating measurements in different laboratories.

The specific rotation angle value for (2*R*,3*R*)-DHQ in ethanol ranges from +12.0° to +21.1° (mean value + 16.6° ± 6.4°) and in acetone from +17.3° to +24.6° (mean value + 21.1° ± 3.7°).

Data on the optical activity of other stereoisomers of DHQ is scarce. For solutions of (2*S*,3*S*)-DHQ in methanol, values of the specific rotation angle equal to −15.0° and −16.2° are given, and in acetone −20.6°. For the methanol solution of (2*R*,3*S*)-DHQ, a single value of −20.0° has been described. From the data in Table 2, it can be stated that in acetone, the modulus of the specific rotation angle value of the stereoisomers of DHQ is higher than in alcohols. It should also be emphasized that the authors do not always provide a full description of the methods for obtaining the optical activity data, which makes it difficult to reproduce the experiment and confirm the results.

Several explanations for the observed heterogeneity in the polarimetry data are possible. One reason is the imperfection of the analysis technique and the human factor. In addition, it may be a result of the presence of accompanying optically active impurities, including other stereoisomers of DHQ.

Therefore, reliance on literature data of polarimetry for the identification of stereoisomers of DHQ is currently impossible. Repeated studies are required in this area, considering the transition to a new technological level and automated analytical equipment.

### 3.2 NMR spectroscopy

In contrast to polarimetry, nuclear magnetic resonance (NMR) spectroscopy is rarely mentioned in pharmacopeial monographs on APIs and remains mainly a tool for researchers. Data on the spectral characteristics of DHQ stereoisomers has accumulated (Table 3).

**TABLE 3:** Bibliographic data on the NMR ^1^H spectroscopy analysis of DHQ stereoisomers.

Stereoisomer	Solvent	Frequency, MHz	Spectral characteristics			Reference
			*δ*, ppm		*J*, Hz	
			H2	H3		
*trans*	MeOH-*d*_*4*_	800	4.93	4.52	12.0	Terekhov et al. (2024b)
		600	4.90	4.49	11.7	Kuspradini et al. (2009)
		500	4.89	4.49	11.4	Yoon et al. (2020)
			5.00	4.60	11.5	Turnbull et al. (2004)
		300	4.90	4.51	11.5	Baderschneider and Winterhalter (2001)
		-	4.91	4.49	11.5	Ohmura et al. (2002)
			4.89	4.57	11.7	Lundgren and Theander (1988)
	Me_2_CO-*d*_*6*_	600	5.02	4.61	11.4	Jiang et al. (2015)
		400	5.04	4.63	11.5	Fedorova et al. (2019)
		-	5.01	4.60	11.3	Kiehlmann and Li (1995)
	Me_2_SO-*d*_*6*_	800	4.96	4.48	11.4	Mohammed et al. (2022)
		300	5.00	4.51	11.2	Braune et al. (2001)
		-	4.99	4.52	11.3	Trofimova et al. (2015)
	-	500	4.98	4.50	11.2	Nifant’ev et al. (2006)
		-	4.98	4.59	11.2	Wang et al. (2012)
*cis*	MeOH-*d*_*4*_	800	5.31	4.20	2.4	Terekhov et al. (2024b)
		500	5.30	4.20	3.0	Turnbull et al. (2004)
	Me_2_CO-*d*_*6*_	-	5.42	4.27	2.8	Kiehlmann and Li (1995)
	-	-	5.29	4.17	2.8	Lundgren and Theander (1988)
			5.28	4.19	2.9	Ohmura et al. (2002)

This method of analysis does not allow for the identification of enantiomers but can differentiate between diastereomers. There is considerably more information on NMR spectra for *trans*-DHQ than for the *cis*-configuration of this flavanonol. Deuterated methanol, and less frequently acetone and dimethyl sulfoxide, are predominantly used for NMR spectroscopy, which can be attributed to the solubility of DHQ.

The *trans*- and *cis*-isomers can be identified by the chemical shift (*δ*) values for the signals of hydrogen atoms at positions 2 and 3 of the pyranone ring and by the spin-spin coupling constants (*J*) between them. The NMR spectrum of *trans*-DHQ is characterized by signals with chemical shift values of 4.96 ± 0.05 ppm and 4.54 ± 0.05 ppm belonging to hydrogen atoms at positions 2 and 3, respectively. For the *cis*-isomer, these parameters were 5.32 ± 0.06 ppm and 4.21 ± 0.04 ppm, respectively.

**FIGURE 4:**
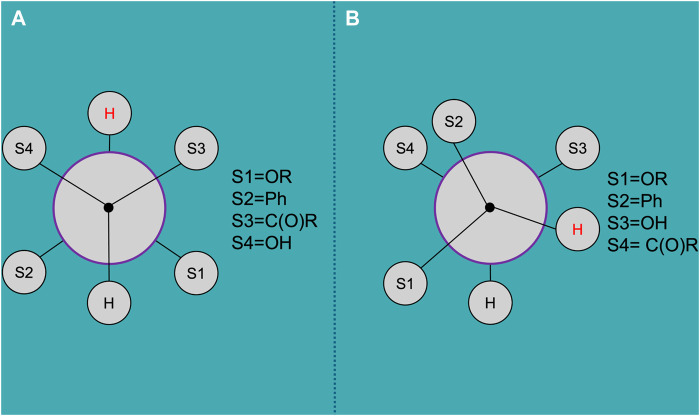
The Newmans projections of DHQ conformation, calculated by NMR data: **(A)** *trans*-DHQ; **(B)** *cis*-DHQ. Created by MestReJ (Navarro-Vázquez et al., 2004).

The spin-spin coupling constants for the DHQ molecules existing in *trans*- and *cis*-configurations were 11.5 ± 0.2 Hz and 2.8 ± 0.2 Hz, respectively. These data can be used to calculate the dihedral angles (*φ*) between hydrogen atoms in positions 2 and 3 for DHQ diastereomers using the Haasnoot-de Leeuw-Altona equation:

$$J=P_{1}\cos^{2}\varphi +P_{2}\cos\varphi +P_{3}+\sum \Delta \chi_{i}(P_{4}+P_{5}\cos^{2}\left(\xi_{i}\varphi +P_{6}\left|\Delta \chi_{i}\right|\right)$$

where *Δχ*_*i*_ is the electronegativity differences of the substituents, *ξ* is the sign parameter takes the value + 1 or −1, depending on the orientation of the substituent, *P*_*1*_, *P*_*2*_, *P*_*3*_, *P*_*4*_, *P*_*5*_, and *P*_*6*_ are empirically derived parameters, which in our case equal to 13.88, −0.81, 0, 0.56, −2.32, and 17.90, respectively (Haasnoot et al., 1980; Coxon, 2009). So, the molecule of *trans*-DHQ is characterized by dihedral angle of 179.0 ± 8.4° between these atoms, while in *cis*-molecule this structural parameter is equal to 68.0 ± 1.5°. The Newmans projections of these conformation are presented in Figure 4. The calculation and visualization were performed in MestReJ [Navarro-Vázquez et al.].

These characteristics allow us to reliably distinguish between the two diastereomers. However, authors of publications often omit details of the methods of analysis, making it difficult to reproduce the results. Enriching the profiles of NMR spectra of DHQ stereoisomers with data from 2D spectroscopy, particularly heteronuclear single-quantum correlation spectroscopy (HSQC) and spectroscopy with the Overhauser effect (NOESY), will help define the structural characteristics of the molecules of the DHQ diastereomers more accurately.

### 3.3 Chromatography

Chromatography allows for the identification and control of the quantity of both the main component and accompanying impurities, making it a more universal analytical method. Semi-preparative chromatography is used for the purification and accumulation of a specific compound. However, information on the chromatography of stereoisomers of DHQ is more modest (Table 4) than in the case of the methods discussed above.

**TABLE 4:** Bibliographic data on the separation of DHQ stereoisomers by chromatography.

Conditions				Characteristics					Reference
Phase		T, °C	Flow rate, mL/min	DHQ isomer	*RT*, min	*NTP*	*Rs*		
Stationary	Mobile								
tris- (4-methylbenzoate) -cellulose	MeCN/H_2_O (10:90→20:80→30:70)_30 min 10 min_	35	0.50	2*S*,3*R*	27.9	20,300	2.6		Elsinghorst et al. (2011)
				2*S*,3*S*	29.9	25,600		2.4	
				2*R*,3*R*	31.6	31,100	2.4		
				2*R*,3*S*	33.5	33,800			
tris- (4-methylbenzoate) -cellulose	MeCN/H_2_O/H_3_PO_4_ (15:85:0.5)	25	0.35	2*S*,3*R*	68.0	2,959*	1.0*		Vega-Villa et al. (2009)
				2*S*,3*S*	73.0	3,411*		1.5*	
				2*R*,3*R*	82.0	2,186*	6.9*		
				2*R*,3*S*	126.0	7,818*			
silica gel with grafted biphenyl radicals	H_2_O (0.1% HCOOH)/MeOH (0.2% HCOOH) (93:7→79:21)_15 min_	60	0.65	*trans*	10.1	20,351	5.3		Terekhov et al. (2024a)
				*cis*	11.6	26,285			

^*^values was calculated by the authors of current review; *RT*, retention time; *NTP*, number of theoretical plates; *Rs* - resolution.

Reversed-phase chromatography was used in all detected methods. In general, the elution conditions are usual for DHQ: acid-modified mobile phase with a major proportion of water and acetonitrile or methanol as an organic solvent.

Due to differences in the physicochemical properties of the diastereomers, *trans*- and *cis*-DHQ can be detected and analysed by high-performance liquid chromatography (HPLC). The separation of DHQ diastereomers using silica gel with grafted biphenyl radicals as a stationary phase has been described. The number of theoretical plates (*NTP*) for *trans*- and *cis*-DHQ was 20,351 and 26,285, respectively, and the peak-to-peak resolution (*Rs*) was 5.3. These data indicates a high efficacy of diastereomers separation, which can be a result of π,π-stacking between the molecules of stationary phase and the flavanonol. The total elution time for a single sample takes 15 min. Thus, the methodology designed for the identification of diastereomers of DHQ appears to be adapted to the needs of the pharmaceutical industry.

The separation of a mixture of enantiomers requires the creation of chiral conditions in a mobile or stationary phase. Tris-(4-methylbenzoate)-cellulose was used as a chiral stationary phase in two articles. Despite good separation efficacy of all four stereoisomers, their retention times (*RT*) remained impressive and required up to 126 min of waiting time, which is unappropriated for routine analysis. Nevertheless, this method is used for preparative separation of DHQ enantiomers.

The observed data indicate good intermediate results in the chromatographic separation of DHQ stereoisomers. However, further research is required to develop techniques to identify DHQ enantiomers more reliably and rapidly.

## 4 Pharmacological aspects

### 4.1 Pharmacodynamics

A number of articles devoted to exploring the biological effects of DHQ are published annually. However, these studies are conducted with no relation to the stereochemical structure of DHQ. In the current review, we tried to accumulate the data that were obtained with respect to the stereoisomerism of this flavonoid and provide an opportunity to compare the affinities of isomers to different biological targets. In the case of *in vivo* or *in vitro* experiments, the configuration of DHQ molecules should be confirmed by any analytical method, e.g., polarimetry. We found only 3 studies that met these criteria (Table 5).

**TABLE 5:** Bibliographic data on the affinities of DHQ stereoisomers to the biological targets.

Type of experiment	Pharmacological effect	Biological target	Descriptor of isomer affinity	Relative* affinities of DHQ isomers, %				Reference
				2*R3R*	2*S*3*S*	2*R*3*S*	2*S*3*R*	
*in silico*	anti-cancer	EGFR	docking score (Schrödinger)	96	98	97	100	Oi et al. (2012)
		PI3-K		89	96	92	100	
*in vitro*	neuroprotective	Aβ42	tioflavin-T fluorescence test	86	100	n/a	n/a	Sato et al. (2013)
*in silico*	anti-viral	M^pro^ of SARS-CoV-2	docking score (Schrödinger)	74	100	n/a	n/a	Fischer et al. (2020)

^*^The highest value of affinity was taken as 100%; EGFR, epidermal growth factor receptor; PI3-K, phosphoinositide 3-kinase; M^pro^, main protease; Aβ42, amyloid β proteins.

This table is quite revealing in several ways. First, the study of differences in pharmacological properties of DHQ stereoisomers is in the early stages: The majority of data were obtained *in silico* and there are no experimental results *in vivo*. Even if authors took into account the stereoisomerism of this flavonoid, the most biology tests performed were conducted with a reduced collection of DHQ enantiomers. So, Oi et al. (2012) designed the research of anti-cancer properties of DHQ in translation form, including the stages *in silico*, *in vitro*, *ex vivo*, and *in vivo*. However, the pure (85%) (2*R*,3*R*)-DHQ was used during the *in vitro* test, and the *in vivo* experiment was performed with a mixture of (2*R*,3*R*)- and (2*S*,3*R*)-isomers, which characterized 90% purity. In another case, Sato et al. (2013) synthesized the racemate of *trans*-DHQ, using the modified Roschek et al. (2009) method, and separated the individual enantiomers. Although the purity of the generated DHQ samples was not provided, the ability to aggregate the 42-residue amyloid β proteins (Aβ42) was assessed for both *trans*-enantiomers. We suppose that the main reason for the observed gap in this research area is the difficulties in obtaining pure samples of DHQ isomers in amounts that would be enough to conduct the *in vivo* study. With that knowledge in mind, the *in silico* analysis looks preferable for the screening of pharmacology activity. However, these should be approved in moist biology experiments (Taldaev et al., 2022B).

What is interesting about the data in this table is that depending on the biological target structure, the difference in DHQ isomers’ affinities may be quite high or absolutely absent. For example, in the case of epidermal growth factor receptor (EGFR) and phosphoinositide 3-kinase (PI3-K), the molecule of DHQ builds in ATP-binding sites by phenolic groups in positions 3′, 4′, and 5. As the pyrocatechinic substitute can freely rotate about σ-bound and the hydroxyl group in position 3 did not take part in supramolecular interaction, the configuration of chiral centers does not affect the affinity of DHQ to these proteins. The similar explanation can be provided in the case of Aβ42. Using computational analysis, Ginex et al. (2018) showed that the covalent binding of the oxidized pyrocatechinic ring with Lys16 plays the primary role in aggregation of this biological target. The inhibition of the main protease (M^pro^) of Severe Acute Respiratory Syndrome CoronaVirus 2 (SARS-CoV-2) demonstrates another situation. Fischer et al. (2020) reported that the DHQ molecule interacts with M^pro^ by phenolic groups in positions 3′ and 7 forming H-bonds with Gln192 and Cys44, respectively. However, the (2*S*,3*S*)-configuration provides an opportunity to generate an additional bound between the alcohol group in position 3 and Glu166 resulting in better affinity of (2*S*,3*S*)-DHQ to M^pro^. These data confirmed that configuration of chiral centers in flavanonol molecule affects on the efficacy of its interaction with protein. This knowlege should be taken into accounte during the development of new chiral drugs.

### 4.2 Pharmacokinetics

Due to the lack of stereoselective analytical methods for the quantitative control of DHQ isomers, the data on pharmacokinetics is poor. The only article on this topic in rats was published in 2009 by Vega-Villa et al. (2009), where the HPLC approach was used. Later, these data were discussed more broadly (Vega-Villa et al., 2011).

The significant impact of stereochemical structure on the pharmacokinetics of DHQ isomers was discussed. The elimination half-life (*t*_*1/2*_) of (2*R*,3*R*)-DHQ was 14.63 ± 2.18 h, and it was at least 1.6 times lower than the value of this parameter for any other enantiomer of this flavonoid. The reason for this result is not explained. Probably, the amount of (2*R*,3*R*)-DHQ was much higher than the other three isomers, the binding by blood proteins was not full, and a significant part of this enantiomer was excreted.

Comparing with *cis*-DHQ, the renal and hepatic clearances of *trans*-DHQ were 22 times lower and 18 times higher, respectively. The 2*R*-configuration was associated with a greater elimination rate. Another interesting observation was the accumulation of DHQ isomers in the flesh and skin. The percentage of *cis*-diastereomers was a greater order than for *trans*-isomers. Furthermore, the 2*S*-configuration was associated with higher accumulation in body tissues.

This study has several limitations. The chromatographic parameters on initial racemic mixture of DHQ were not presented. The AUC data reported in both articles are contradictory. As mentioned by Vega-Villa et al. (2011), the pharmacokinetics of DHQ was not adequately researched, as the sampling period was not enough to assess exposure. Additionally, the biochemistry of rats and humans differs significantly, so it is not appropriate to transfer data from one species to another. Taken together, these limitations indicate the necessity of further research on the pharmacokinetics of DHQ stereoisomers.

## 5 Conclusion

The present review was undertaken to clarify the current state of DHQ stereoisomerism in pharmaceutical science. In the course of the work, it was found that due to the heterogeneity of data on the optical activity of flavanonol molecules, it is difficult to rely on the results of polarimetry when conducting research and developing regulatory documentation. The information on NMR spectroscopy of DHQ diastereomers is more reliable; however, this method does not provide an opportunity to identify individual stereoisomers and has not been implemented in routine pharmaceutical analysis. Of all the methods considered, only HPLC is currently characterized by sufficient enantioselectivity. The wide variety of identified biological effects of DHQ in the absence of information about the substance and evidence of its stereoisomeric composition does not give confidence in the effectiveness and safety. The results of the conducted review of scientific literature indicate the necessity of revision in the pharmacology of DHQ taking into account its stereoisomerism.
